# A Shadow-Test Approach to Adaptive Item Calibration

**DOI:** 10.1007/s11336-020-09703-8

**Published:** 2020-06-17

**Authors:** Wim J. van der Linden, Bingnan Jiang

**Affiliations:** 1 University of Twente, Enschede, The Netherlands; 2 ACT, Inc., Iowa City, IA USA

**Keywords:** adaptive testing, Bayesian $D_{\mathrm{s}}$-optimality, Gibbs sampling, item calibration, item response models, MCMC algorithm, shadow-test approach

## Abstract

A shadow-test approach to the calibration of field-test items embedded in adaptive testing is presented. The objective function used in the shadow-test model selects both the operational and field-test items adaptively using a Bayesian version of the criterion of $D_{\mathrm{s}}$-optimality. The constraint set for the model can be used to hide the field-test items completely in the content of the test as well as to deal with such practical issues as random control of their exposure rates. The approach runs on efficient implementations of the Gibbs sampler for the real-time updating of the ability and field-test parameters. Optimal settings for the proposed algorithms were found and used to demonstrate item calibration with smaller than traditional sample sizes in runtimes fully comparable with conventional adaptive testing.

## Introduction

The current focus of adaptive testing programs typically is on the selection of items from an operational pool with optimization of the estimates of the examinees’ ability parameters as statistical objective. As for the item pool, it is not uncommon for it to be calibrated with response data collected in separate field-test studies with the items organized as sets of linked test forms administered to volunteering examinees. This type of field-test studies requires considerable resources. It may also run into such problems as less representative data due to lack of the operational testing conditions, danger of early security breaches, detection of misfitting items only after all data have been collected, and drifting of the item parameters from the scales for the current item pool due to accumulated linking errors.

An alternative approach is to embed a few field-test items directly in the operational adaptive tests for each of the examinees. A unique advantage possible for this approach is adaptive selection of the items capitalizing on the real-time updates of the examinees’ ability parameters during testing. Just as adaptive selection of the operational items leads to a considerable reduction of the test length necessary to score the examinees, similar selection of the field-test items can be expected to lead to substantial reduction of the sample size necessary to calibrate new items, an advantage already demonstrated in a few recent item calibration studies (Ren et al. 2017; van der Linden and Ren 2015). Other possible advantages of embedded field-testing of new items are more practical and include saving the time and expenses necessary to run separate calibration studies, collection of responses from motivated examinees answering the items under operational conditions, possibility of real-time monitoring of item fit and early withdrawal of items that appear to be malfunctioning, and estimation of all item parameters directly on the scales in use for the item pool.

These advantages are only possible when we have algorithms that manage the processes of parameter updating and optimal item selection both for the operational and field-test items in real time. Also, some additional practical issues need to be resolved. For example, the identity of the field-test items should be hidden from the examinees. If they would be able to find out which items do and do not add to their scores, they may take the latter less seriously and even decide to answer them just randomly to save time for the items that do count. Also, as field-test items are now exposed to their future population of examinees prior to operational testing, it may be necessary to prevent their possible compromise due to this extra exposure. An obvious option is to subject them to the same type of shadow-test-based exposure control as for the operational items in the pool (van der Linden and Choi 2019) but with adjusted values for their target exposure rates.

The goal of the research in this report was to design and optimize the algorithms required for real-time adaptive testing with embedded item calibration. The approach is derived from a more general description of adaptive testing programs as multiple, statistical and non-statistical processes with real-time updating of all intentional statistical parameters in van der Linden (2018). The tools available to run these processes are sequential Bayesian parameter updating, application of statistical optimal design theory to plan each next data collection step, and use of the shadow-test approach to manage all processes simultaneously and keep each of the non-statistical parameters of the program within their required bounds.

The basic setup of the testing program considered in the current research was an adaptive test with a few field-test items administered to each of the examinees in positions randomly selected near the end of the test (for example, three of the last five). The choice of setup was motivated by the necessity to compromise between the ideal of selecting the field-test items capitalizing on the statistical information offered by the posterior distributions of the examinees’ ability parameters and the requirement to hide their identity to have them taken equally seriously as the operational items in the test. As explained below, the identity of the field-test items can further be hidden by embedding them fully in the test content. The compromise between the wish to have maximum statistical information and to hide the identity of the field-test items may need to be extended to account for the effects of possible fatigue or speededness of the test as well, an issue reserved for future research.

The following sections introduce the response model used as an example in the research, explain each of the three tools referred to above in more detail, and present results from studies conducted to optimize the settings of the algorithms and demonstrate their application to a real-world adaptive testing program. All results point at item calibration with smaller sample sizes than for separate calibration studies obtained in runtimes fully comparable with those for current maximum-information adaptive testing without any field testing of new items.

## Response Model

Let $i=1,\ldots ,I$ denote the operational items and $f=1,\ldots ,F$ the field-test items in the pool. Both categories of items are scored by response variables *U*$_{i}\in \{0,1\}$ and *U*$_{{f}}\in \{0,1\}$, respectively. As an example, the operational items are assumed to be calibrated and their fit checked under the three-parameter logistic (3PL) model, which explains the probability of a correct response on each of the items as

1 $$\begin{aligned} \Pr \{U_{i}=1|\theta ,a_{i},b_{i},c_{i}\}\equiv p(\theta ;a_{i},b_{i},c_{i})\equiv c_{i}+(1-c_{i})\frac{\exp [a_{i}(\theta -b_{i})]}{1+\exp [a_{i}(\theta -b_{i})]}, \end{aligned}$$

where $b_{i}\in \mathbb {R}$ and $a_{i}\in \mathbb {R}^{+}$ can be interpreted as parameters for the difficulty and discriminating power of item *i*, respectively, and $c_{i}\in (0,1]$ as the probability of a correct response to the item resulting from purely random guessing. The model allows us to write the probability function of the response distribution for each examinee–item combination as

2 $$\begin{aligned} f(u_{i}|\theta ,{\varvec{\xi }}_{i})\equiv p(\theta ,{\varvec{\xi }}_{i})^{u_{i}}[1-p(\theta ,{\varvec{\xi }}_{i})]^{1-u_{i}}, \end{aligned}$$

where ${\varvec{\xi }}_{i}\equiv (a_{i},b_{i},c_{i}).$ The field-test items are calibrated under the same model. It is prudent to monitor their fit to the response model permanently during the calibration process to prevent the examinees from having to spend unnecessary time on malfunctioning items. The question of how to adjust goodness-of-fit statistics for application in real-time monitoring is currently investigated.

One of the novel aspects of the approach outlined below is that, rather than storing fixed point estimates for all parameter in the adaptive testing system, each of them is permanently represented by a short vector of draws from its most recent posterior distribution. Due to this decision, all calculations during operational testing simplify with results that automatically account for the uncertainty about any of the parameters. The best way to collect these draws during initial item pool calibration is through Bayesian estimation with Markov chain Monte Carlo (MCMC) sampling of the posterior distributions of the parameters. Alternatively, for an existing item pool, we could use an asymptotic argument sampling normal distributions with the point estimates and standard errors of the item parameters as mean and standard derivation. This is a one-time requirement only, though. Once the process of embedded item calibration begins, the system automatically generates new items ready for operational use along with appropriate vectors of posterior draws for each of them.

## Sequential Bayesian Parameter Updating

During testing, two distinct processes of parameter updating are to be managed, one for the ability parameters of the examinees and the other for the parameters of the field-test items. These parameters are the intentional parameters of the two processes. In addition, both processes have nuisance parameters. At the update of an ability parameter after a response to an operational item, the parameters of the item are nuisance parameters. But when the parameters of a field-test item are updated, the ability parameter of the examinee that produced the response changes its status from intentional to nuisance parameter. The status of parameters as nuisance parameters does not mean that we can ignore them; just as for the intentional parameters, we need to fully account for their impact on the responses that are collected. Neither is it correct to substitute point estimates for them; doing so would lead to loss of optimality of the criteria for the selection of the next operational and field-test items, especially early on in the two processes when the uncertainty about the ability and field-test parameters is maximal (for an example of the initial uncertainty about latter, see the first lines in Figs. 2, 3, 4).

The appropriate way to update intentional parameters in a continuous processes of response collection is through sequential use of Bayes theorem integrating out each of nuisance parameters. In the current context, after the* k*th operational item in the test and response vector $\varvec{u}_{k}=(u_{1},\ldots ,u_{k})$, the theorem is used to update the marginal posterior distribution of the ability parameter from *f*($\theta |\varvec{u}_{k-1}$) to *f*($\theta |\varvec{u}_{k}$). The prior distributions for the operational parameters $\varvec{\xi }_{k}$ during the update are their posterior distributions obtained during item pool calibration. As these distributions do not depend on any data collected during testing, we have prior independence of $f(\theta \mid \varvec{u}_{k-1})$ and $f(\varvec{\xi }_{k})$. Besides, the standard assumption of local independence implies $f(u_{k}\mid \theta ,\varvec{u}_{k-1})=f(u_{k}\mid \theta )$. Hence, the application of Bayes theorem, in its regular form equal to

$$\begin{aligned} f(\theta \mid u_{k})=\frac{\int f(u_{k}\mid \theta ,\varvec{\xi }_{k})f(\theta ,\varvec{\xi }_{k}\mid \varvec{u}_{k-1})\mathrm{d}{\varvec{\xi }}_{k}}{\int \int f(u_{k}\mid \theta ,\varvec{\xi }_{k})f(\theta ,\varvec{\xi }_{k}\mid \varvec{u}_{k-1})\mathrm{d}\theta \mathrm{d}{\varvec{\xi }}_{k}}, \end{aligned}$$

simplifies to

3 $$\begin{aligned} f(\theta |\varvec{u}_{k})=\frac{f(u_{k}|\theta )}{f(u_{k})}f(\theta |\varvec{u}_{k-1}), \end{aligned}$$

where

4 $$\begin{aligned} \frac{f(u_{k}|\theta )}{f(u_{k})}=\frac{\int f(u_{k}|\theta ,\varvec{\xi }_{k})f(\varvec{\xi }_{k})\mathrm{d}\varvec{\xi }_{k}}{\int \int f(u_{k}|\theta ,\varvec{\xi }_{k})f(\varvec{\xi }_{k})f(\theta |\varvec{u}_{k-1})\mathrm{d}\theta \mathrm{d}\varvec{\xi }_{k-1}}. \end{aligned}$$

and $f(u_{k}|\theta ,\varvec{\xi }_{k})$ is the examinee’s response probabilities in (2) for the *k*th item in the test. Observe how nicely (3) factors the posterior density of $\theta$ into the product of one factor depending on the new response and a second summarizing the information about it contained in all earlier responses.

Likewise, the update of the joint posterior distribution of field-test parameter $\varvec{\xi }_{{f}}$ upon the *l*th administration of the item is

5 $$\begin{aligned} f(\varvec{\xi }_{{f}}|\varvec{u}_{l})=\frac{f(u_{l}|\varvec{\xi }_{{f}})}{f(u_{l})}f(\varvec{\xi }_{{f}}|\varvec{u}_{l-1}), \end{aligned}$$

where

6 $$\begin{aligned} \frac{f(u_{l}|\varvec{\xi }_{{f}})}{f(u_{l})}=\frac{\int f(u_{l}|\theta _{l},\varvec{\xi }_{{f}})f(\theta _{l}|\varvec{u}_{k'})\mathrm{d}\theta _{l}}{\int \int f(u_{l}|\theta _{l},\varvec{\xi }_{{f}})f(\varvec{\xi }_{{f}}|\varvec{u}_{l-1})f(\theta _{l}|\varvec{u}_{k'})\mathrm{d}\theta _{l}\mathrm{d}\varvec{\xi }_{{f}}}, \end{aligned}$$

$k'$ is the number of operational items already answered by the current examinee, and $f(u_{l}|\theta _{l},\varvec{\xi }_{{f}})$ now is the response probability of the examinee in (2) for field-test item *f*.

For both processes of parameter updating we thus have a simple recurrence relation between the new and previous posterior density, with the latter serving as the new prior. In spite of their apparent simplicity, because of the presence of multiple integrals in (4) and (6), both relations may seem computational demanding. However, as is well known now, use of Monte Carlo sampling from the joint posterior distribution of all parameters avoids having to calculate any of these integrals (e.g., Gelman et al. 2014, Part 3; Gilks et al. 1996). Particularly attractive is the use of the Gibbs sampler which, without any further assumptions, allows for an extremely efficient implementation in the current context of adaptive testing.

Recall that the parameters of the operational items are represented in the system by vectors of posterior draws collected during their calibration. The Gibbs sampler capitalizes on their presence in the first of the following two updates:

**Update of Ability Parameter**$\theta$

1. The sampler iterates between resampling of the vectors of draws for the parameters $\varvec{\xi }_{k}$ of the last operational item administered to the examinee and a Metropolis–Hastings (MH) step to sample from the new distribution of the $\theta$ parameter.
2. When the sampler is stopped, the current vector of draws for $\theta$ in the system is overwritten with a selection of new draws collected from the stationary part of the Markov chain.

**Update of Field-Test Parameters**$\varvec{\xi }_{{f}}$

1. The sampler now iterates between resampling of the last vectors of draws for the $\theta$ parameter of the current examinee and an MH step to sample the new distribution of field-test parameter $\varvec{\xi }_{{f}}$.
2. When the sampler is stopped, the current vector of draws for $\varvec{\xi }_{{f}}$ in the system is overwritten with a selection of new draws collected from the stationary part of the Markov chain.

The sequential nature of both updates permits a natural choice of proposal and prior distributions for their MH steps. For instance, for the update of the $\theta$ parameter, let ($\theta _{k-1}^{(1)},\ldots ,\theta _{k-1}^{(S)}$) be the vector of *S* posterior draws currently present in the system, which has mean and variance

7 $$\begin{aligned} \mu _{k-1}=S^{-1}\sum _{s=1}^{S}\theta _{k-1}^{(s)} \end{aligned}$$

and

8 $$\begin{aligned} \sigma _{k-1}^{2}=S^{-1}\sum _{s=1}^{S}\left( \theta _{k-1}^{(s)}-\mu _{k-1}\right) ^{2}, \end{aligned}$$

respectively. As the MH steps require specification of the prior density of each of the values of $\theta$ drawn from the posterior distribution, which is known to converge to normality anyhow (Chang and Ying 2009), an obvious choice is

9 $$\begin{aligned} f_{k-1}(\theta \mid \varvec{u}_{k-1})\equiv N(\mu _{k-1},\sigma _{k-1}^{2}). \end{aligned}$$

Likewise, for the proposal distribution at iteration steps $r=1,\ldots ,R$, an effective choice is

10 $$\begin{aligned} q_{k}(\theta \mid \theta ^{(r-1)})\equiv N(\theta ^{(r-1)},\sigma _{k-1}^{2}). \end{aligned}$$

Because of the symmetry of (10), upon the *r*th draw for $\varvec{\xi }_{k}$, the draw for the $\theta$ parameter simplifies to

1. draw candidate value $\theta ^{(c)}$ for $\theta ^{(r)}$ from the proposal distribution in (10);
2. accept $\theta ^{(r)}=\theta ^{(c)}$ with probability
  11 $$\begin{aligned} \min \left\{ \frac{N(\theta ^{(c)}\mid \mu _{k-1},\sigma _{k-1}^{2})f(u_{k};\theta ^{(c)},{\varvec{\xi }}_{k}^{(r)})}{N(\theta ^{(r-1)}\mid \mu _{k-1},\sigma _{k-1}^{2})f(u_{k};\theta ^{(r-1)},{\varvec{\xi }}_{k}^{r-1})},1\right\} ; \end{aligned}$$
  otherwise $$\theta ^{(r)}\equiv \theta ^{(r-1)}$$.

As the Markov chain is already on target right from its start for each of the nuisance parameters, convergence for the single intentional parameter is extremely fast. Also, the only quantity that needs to be calculated to evaluate (11) is the product of a normal density and the response probability for the last item in its numerator (the denominator was already calculated in the previous step). Finally, it is not necessary to tune the proposal distribution, typically a time-consuming task. The distribution automatically follows the posterior distribution with a somewhat greater variance, known to be an efficient choice for low-dimensional parameters (Gelman et al. 2014, sect. 12.2). For further details, such as the optimal choice of burn-in length, autocorrelation estimates, and control of Monte Carlo error, the reader should consult the optimization study of the sampler for application in regular adaptive testing without any item calibration in van der Linden and Ren (2020).

The sampler for the updates of each of the field-test parameters added in this research follows the same setup as in (7)–(11). The only difference is a temporary change of their scale to support the normality of (9) and (10). Prior to the update, the parameters are transformed to

$$\begin{aligned} b_{{f}}^{*}\equiv & {} b_{{f}}\\ a_{{f}}^{*}\equiv & {} \text {ln}(a_{{f}})\\ c_{{f}}^{*}\equiv & {} \text {logit}(c_{{f}}) \end{aligned}$$

with back transformation to the original scale immediately after the update. The authors are aware of the fact that, for the multi-parameter case, direct sampling from the joint posterior distribution is generally more efficient. But, given the already extremely fast convergence, the gains for the current application have been found to be too small to justify the efforts.

## Optimal Design Criteria

The ultimate goal of the two processes of adaptive testing and item calibration is maximum information about their intentional parameters. To realize this, sequential updating of these parameters is not sufficient. The necessary additional step is selection of each next item to have the best possible match between the intentional and nuisance parameters for the pertinent process.

Criteria for such matches can be derived from statistical optimal design theory. Among its many criteria, the more favorable belong to the class of $D_{\mathrm{s}}$-optimality; that is, the choice of a minimum determinant of the covariance matrix of the estimators of the intentional parameters given the nuisance parameters. For a general introduction to this class of criteria from a frequentist perspective, refer to Holling and Schwabe (2018), while Berger (2018) should be consulted for a general introduction to optimal item-calibration design. Alternative optimal design criteria for use in adaptive item calibration have been studied by Ren et al. (2017) and van der Linden and Ren (2015) but these authors also found the criterion of $D_{\mathrm{s}}$-optimality to serve its purpose generally best. Minimization of the determinant of the covariance matrix for maximum-likelihood estimators is (asymptotically) equivalent to maximization of their Fisher information matrix. As the items are selected in a continuous process of data collection, the proposed version of the $D_{\mathrm{s}}$-criterion is selection of items with the maximum marginal profit for either of these determinants.

For $\theta$ as intentional parameter, the information matrix reduces to a scalar (equal to its “determinant”) widely used as item-selection criterion in conventional adaptive testing. For the 3PL model, it is easily obtained as

12 $$\begin{aligned} I(\theta ;{\varvec{\xi }}_{i})=a_{i}^{2}\frac{1-p(\theta ;{\varvec{\xi }}_{i})}{p(\theta ;{\varvec{\xi }}_{i})}\left( \frac{p(\theta ;{\varvec{\xi }}_{i})-c_{i}}{1-c_{i}}\right) ^{2}. \end{aligned}$$

Because of local independence, (12) is additive in the items and consequently all contributions by the earlier items can be ignored when selecting the next. However, as both $\theta$ and $\varvec{\xi }_{i}$ are known only through their posterior distributions, the proper way of using (12) is by first taking its expectation across these distributions. The proposed Gibbs sampler simplifies the calculation of the expectation considerably. In addition to the vector $(\theta ^{(1)},\ldots ,\theta ^{(S)}$) of draws from last update of $\theta$, let $({\varvec{\xi }}_{i}^{(1)},\ldots ,{\varvec{\xi }}_{i}^{(S)})$ be the vector for the parameters of operational item *i* permanently present in the system. Using these draws, the posterior expected information for *i* as candidate item can be calculated as

13 $$\begin{aligned} \int \int I(\theta ;\varvec{\xi }_{i})f(\theta \mid u_{k})f(\varvec{\xi }_{i})\mathrm{d}\theta \mathrm{d}\varvec{\xi }_{i}\thickapprox S^{-1}\sum \limits _{s=1}^{S}I(\theta ^{(s)},{\varvec{\xi }}_{i}^{(s)}). \end{aligned}$$

The length of both vectors of draws is taken to be equal here for notational convenience only. For vectors of different lengths, an efficient choice is to recycle the shorter against the longer. The length of the vectors directly controls the Monte Carlo error in (13), an issue further addresses below.

As for the selection of the field-test items, the Fisher information matrix for field-test item *f* is equal to

14 $$\begin{aligned} I(\varvec{\xi }_{{f}};\theta )=\left( -\frac{\partial ^{2}}{\partial \xi _{j}\partial \xi _{j'}}\text {ln}(f(u;\theta ,\varvec{\xi }_{{f}})\right) ,\quad j,j'\in \{1,2,3\}. \end{aligned}$$

Using

$$\begin{aligned} \lambda _{{f}}\equiv \frac{(1-p_{{f}})}{p_{{f}}(1-c_{{f}})^{2}} \end{aligned}$$

with $p_{{f}}\equiv f(u;\theta ,\varvec{\xi }_{{f}})$, the elements of (14) are easily obtained from

15 $$\begin{aligned}&\frac{\partial ^{2}}{\partial a_{{f}}^{2}}\text {ln}(f(u;\theta ,\varvec{\xi }_{{f}}))=-(p_{{f}}-c_{{f}})^{2}(\theta -b_{{f}})^{2}\lambda _{{f}}, \end{aligned}$$

16 $$\begin{aligned}&\frac{\partial ^{2}}{\partial b_{{f}}^{2}}\text {ln}(f(u;\theta ,\varvec{\xi }_{{f}}))=-a_{{f}}^{2}(p_{{f}}-c_{{f}})^{2}\lambda _{{f}}, \end{aligned}$$

17 $$\begin{aligned}&\frac{\partial ^{2}}{\partial c_{{f}}^{2}}\text {ln}(f(u;\theta ,\varvec{\xi }_{{f}}))=-\lambda _{{f}}, \end{aligned}$$

18 $$\begin{aligned}&\frac{\partial ^{2}}{\partial a_{{f}}b_{{f}}}\text {ln}(f(u;\theta ,\varvec{\xi }_{{f}}))=a_{{f}}(p_{{f}}-c_{{f}})^{2}(\theta -b_{{f}})\lambda _{{f}}, \end{aligned}$$

19 $$\begin{aligned}&\frac{\partial ^{2}}{\partial a_{{f}}c_{{f}}}\text {ln}(f(u;\theta ,\varvec{\xi }_{{f}}))=-(p_{{f}}-c_{{f}})(\theta -b_{{f}})\lambda _{{f}}, \end{aligned}$$

20 $$\begin{aligned}&\frac{\partial ^{2}}{\partial b_{{f}}\partial c_{{f}}}\text {ln}(f(u;\theta ,\varvec{\xi }_{{f}}))=a_{{f}}(p_{{f}}-c_{{f}})\lambda _{{f}}. \end{aligned}$$

Though the matrix is still additive in the items, its determinant is not. As a result, the expected contribution by the current examinee to each of the candidate items is no longer independent of its history. This fact seems to complicate evaluation of the $D_{\mathrm{s}}$-criterion for field-test item selection: Using it for the information matrix would require calculation of the posterior expectation of the determinant of the sum of these matrices for each candidate item across the ability parameters of all examinees who have already seen it minus the determinant for the current examinee. On the other hand, direct use of the posterior covariance matrix seems computationally challenging too. It would require simulation of the adaptive test one item ahead for each of the candidate items, running the Gibbs sampler, calculating the determinant of the covariance matrix from the new posterior samples for the parameters of the items, and subtracting the determinant calculated from their current samples. However, the equivalence of the two approaches allows us to capitalize on the data about their key quantities already available in the system.

Let Cov($\varvec{\xi }_{{f}}$) denote the covariance matrix calculated from the last update of the posterior draws for field-test parameters $\varvec{\xi }_{{f}}$ and $I(\varvec{\xi }_{{f}};\theta )$ the information matrix for the current examinee and candidate item *f* as defined by (14)–(20). It is possible to write the criterion as

21 $$\begin{aligned} D_{\mathrm{s}}(\varvec{\xi }_{{f}})=\text {det}\left( \text {Co}\text {v}^{-1}(\varvec{\xi _{\text {f }}})+I(\varvec{\xi }_{{f}};\theta )\right) -\text {det}\left( \text {Cov}^{-1}(\varvec{\xi }_{{f}})\right) , \end{aligned}$$

while its posterior expected value can be calculated using

22 $$\begin{aligned} D_{\mathrm{s}}(\varvec{\xi _{{f}}})\thickapprox S^{-1}\sum _{s=1}^{S}\left[ \text {det}\left( \text {Co}\text {v}^{-1}(\varvec{\xi }_{{f}})+I(\varvec{\xi }_{{f}}^{(s)};\theta ^{(s)})\right) -\text {det}\left( \text {Co}\text {v}^{-1}(\varvec{\xi }_{{f}})\right) \right] . \end{aligned}$$

### Shadow-Test Approach

In the shadow-test approach to adaptive testing (van der Linden and Reese 1998; van der Linden 2005, chap. 9), a full-length shadow test is re-assembled prior to the selection of each item. During each re-assembly, the test must satisfy the following requirements: (i) its objective function guarantees maximum information about the intentional parameters at their current update; (ii) the constraint set covers all test specifications in force for the program; and (iii) all items already administered to the examinee are included in the test. The next item administered to the examinee is the most informative free item in the current shadow test. The examinee only sees these items; all other items remain unknown (hence the name of “shadow test”). A distinct advantage of the approach is optimality of item selection given the constraint set along with guaranteed satisfaction of the latter. A flexible way of implementing the approach is through mixed integer programming (MIP) modeling of the objective function and constraint set with submission of the optimization model to a MIP solver prior to the selection of each item. The solver then returns the IDs of the optimal selection of items, from which the testing algorithm picks the best item for administration. Powerful MIP solvers, fully up to their use in real-time shadow-test assembly, are available as open-source or commercial software programs; information about the runtimes in the empirical studies for the solver used in this research is given below.

Though the approach has only been used for conventional adaptive testing so far, it is actually much more powerful. The objective function and constraint set in the shadow test model can be chosen to manage almost every combination of processes involved in adaptive testing. To do so, we just need to consult the rich body of knowledge and experience present in the field of MIP modeling, especially in its applications to problems of multi-objective decision-making (e.g., Chen et al. 2010; Williams 2013). The remainder of this section illustrates the power of the approach for the simultaneous processes of adaptive testing and item calibration.

Each shadow test is assumed to have $n_{\text {o}}$ operational and $n_{{f}}$ field-test items. Binary variables $x_{i}=0,1$ and $x_{{f}}=0,1$ are used to select the two categories of items from the pool. In addition, we use $S_{k-1}$ to denote the set of indices of the $k-1$ operational and field-test items already administered to the examinee when assembling the *k*th shadow test. Examples of constraints available to deal with possible specifications of the required distributions of quantitative and categorical attributes of the items in the adaptive test are given. Quantitative attributes are attributes with numerical values *q* that represent such features as their response model parameters, expected response times, exposure rates, etc. The distributions of these attributes are required to be constrained by upper and/or lower bounds $b_{q}$. Categorical attributes partition the item pool into subsets $V_{c}$ with common features $c=1,\ldots ,C$. The number of items selected from these subsets are to be constrained by upper and/or lower bounds $n_{c}$. A prime example of a categorical attribute is a (possibly multi-level) classification of the content of the items in the pool; other examples are item type, format, answer keys, etc. Our last example of a possible constraint is a set of items V$_{e}$ that have to be excluded from the test. One possible use of such sets is item-exposure control through random exclusion of items from the shadow tests for the examinees with probabilities controlled by the empirical exposure rates of the items in the pool.

All these bounds and sets are instances of what we have referred to earlier as non-statistical parameters of an adaptive testing program that need to be maintained during testing. From the point of view of test validity, they are more important than its statistical parameters. An example of a shadow-test model for the optimal selection of the *k*th item in the test that does maintain the desired values of these parameters is

23 $$\begin{aligned} \text {maximize}\;\sum _{i=1}^{I}I_{i}(\theta )x_{i}+\sum _{f=1}^{F}D_{\mathrm{s}}(\varvec{\xi }_{{f}})x_{{f}}&(\text {objective function}) \end{aligned}$$

subject to

24 $$\begin{aligned} \sum _{i=1}^{I}x_{i}=n_{\text {o}},&(\#\text { of operational items)} \end{aligned}$$

25 $$\begin{aligned} \sum _{f=1}^{F}x_{{f}}=n_{{f}},&(\#\text { of field-test items)}\end{aligned}$$

26 $$\begin{aligned} \sum _{i,f\in S_{k-1}^{\text {}}}(x_{i}+x_{{f}})=k-1,&\text {(items already administered)}\end{aligned}$$

27 $$\begin{aligned} \sum _{i=1}^{I}q_{i}x_{i}\lesseqqgtr b_{\text {o}q},&\text {(quantitative constraint)}\end{aligned}$$

28 $$\begin{aligned} \sum _{f=1}^{F}q_{{f}}x_{{f}}\lesseqqgtr b_{fq},&\text {(quantitative constraint)}\end{aligned}$$

29 $$\begin{aligned} \sum _{i\in V_{c}}x_{i}\lesseqqgtr n_{\text {o}c},&c=1,\ldots ,C,&\text {(categorical constraints)}\end{aligned}$$

30 $$\begin{aligned} \sum _{f\in V_{c}}x_{{f}}\lesseqqgtr n_{fc},&c=1,\ldots ,C,&\text {(categorical constraints)}\end{aligned}$$

31 $$\begin{aligned} \sum _{i,f\in V_{e}}(x_{i}+x_{{f}})=0,&\text {(exclusion constraint)}\end{aligned}$$

32 $$\begin{aligned} x_{i}=0,1,&i=1,\ldots ,I,&\text {(binary decision variables)}\end{aligned}$$

33 $$\begin{aligned} x_{{f}}=0,1,&f=1,\ldots ,F.&\text {(binary decision variables)} \end{aligned}$$

The objective function simultaneously maximizes the posterior expected information in the shadow test about the examinee’s ability parameter and the field-test parameters in the pool as calculated by the two criteria in (13) and (22). The first two constraints control the numbers of items to be administered. The constraint in (26) effectively sets the decision variables of the $k-1$ operational and field-test items already answered by the examinee equal to one. The next two types of constraints are to maintain bounds on quantitative item attributes. Typically, multiple versions of these constraints are necessary both with upper and/or lower bounds. The same holds for the constraints on the categorical item attributes in (29) and (30). Obviously, the zero bound in (31) prevents the items in set $V_{e}$ in the pool from being administered to the examinee, for instance, because they contain clues to each other (“enemy items”). The last two sets of constraints are necessary to define the decision variables as binary.

**Fig. 1:**
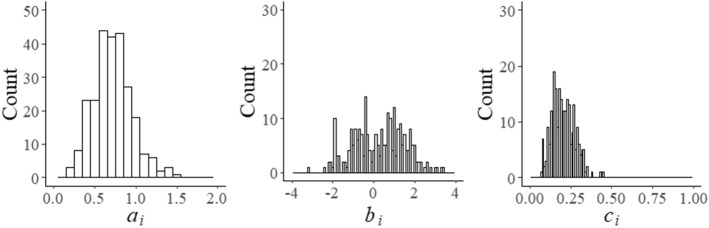
Distributions of parameters of operational items in the pool

**Fig. 2:**
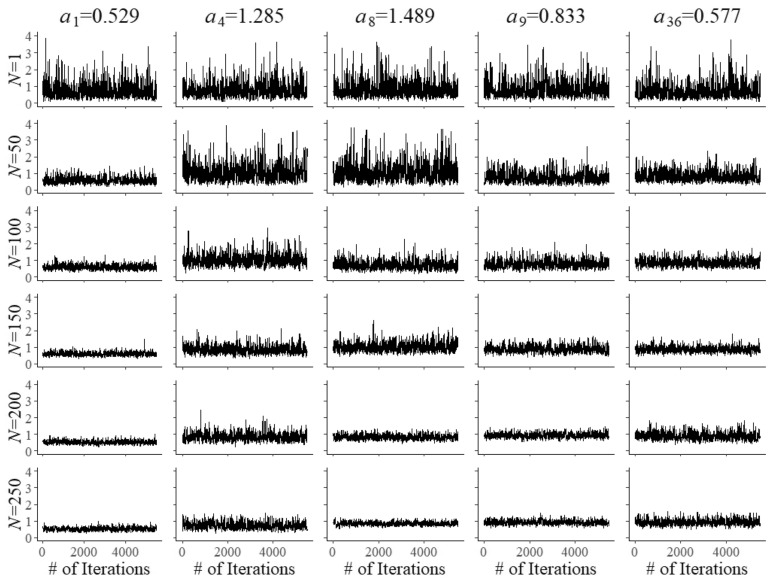
Trace plots of Markov chains for field test parameters $a_{{f}}$ of five arbitrary items after $N=1, 50, 10, 50, 150, 200$, and 250 responses

The model only serves as an example and does not exhaust all possibilities. A major missing type of constraint are those for logical item selection. These constraints are necessary, for instance, to select set-based items along with their stimuli or prevent the selection of items with an enemy relationship. For these and other modeling options, refer to van der Linden (2005). Also, it is important to note that the only required update of this model prior to moving to the next item is addition of the index of the item just administered to set $S_{k-1}$ in the left-hand side of (26) along with an increase of item count *k* in its right-hand side by one.

A key feature of the model in (23)–(33) is complete separation of the optimization of the selection of the operational and field-test items. Though the two problems are controlled by a single model for the shadow test, both have their own objective of maximum information and are subjected to their own bounds. The only difference between the two types of items emerges when an item is selected for administration. When the system arrives at a position in the test reserved for item calibration, it is the currently best field-test item in the shadow test that is administered; otherwise, it is the best operational item. It is possible to combine pairs of constraints as in (27)–(30) replacing them with single versions running across both types of items in the pool. The effect is less specific control of the composition of the shadow test, which may be a disadvantage if there exists a shortage of items with certain combinations of attributes and their calibration has to be accelerated. Also, as operational and field-test items then become exchangeable with respect to the attribute in their common constraint, the system may select an item with the attribute from one of these two categories whereas it may have picked one from the other in case of separate constraints (subject to all other constraints, of course).

**Fig. 3:**
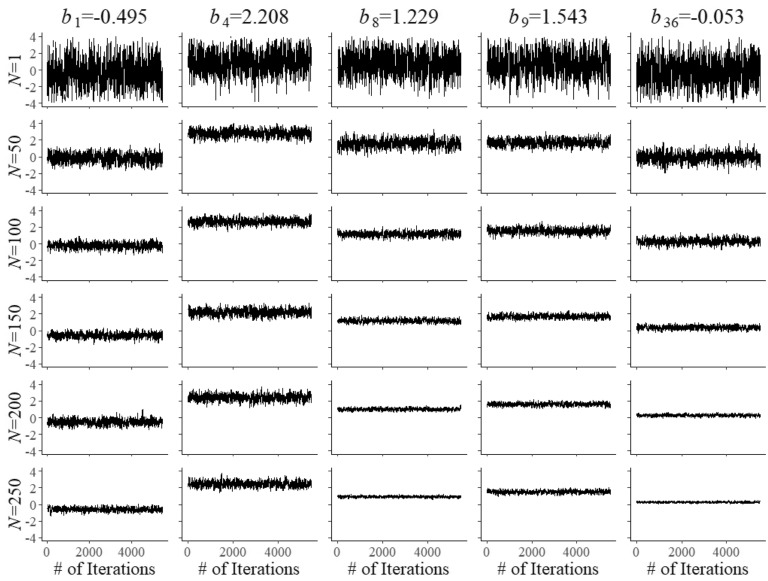
Trace plots of Markov chains for field test parameters $b_{{f}}$ of five arbitrary items after $N=1$, 50, 10, 50, 150, 200, and 250 responses

**Fig. 4:**
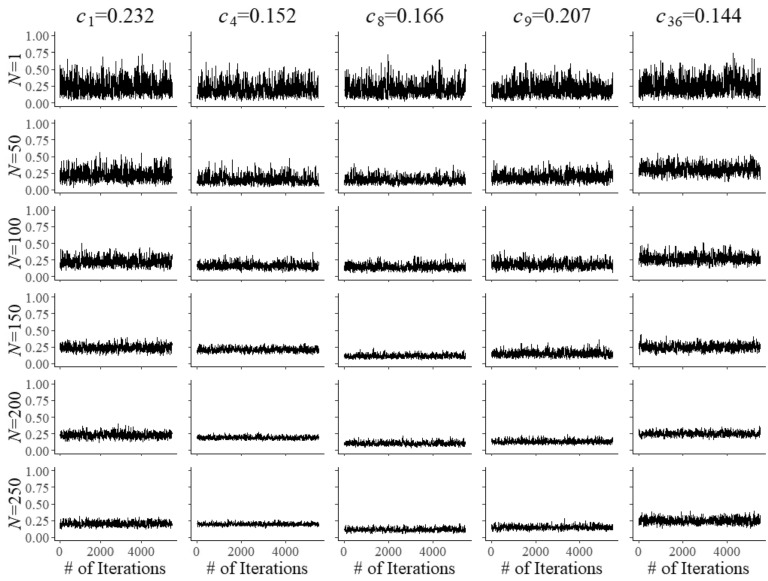
Trace plots of Markov chains for field test parameters $c_{{f}}$ of five arbitrary items after $N=1$, 50, 10, 50, 150, 200, and 250 responses

## Simulation Study

The goal of the study was to explore optimal settings for the proposed shadow-test approach to embedded item calibration and demonstrate its feasibility for real-world application. More specifically, our goal was to address such issues as the best burn-in length of the Markov chain for the updates of the field-test parameters, estimation of its autocorrelation, selection of the sample size of the post burn-in draws for operational use, as well as the recording of the runtimes for our best combination of settings. In order to demonstrate practical feasibility, the combination was evaluated both for its speed of item calibration and the statistical qualities of the item parameter estimates.

### Item Pool

The item pool consisted of a random sample of 250 retired items from a real-world testing program calibrated under the 3PL model. In addition, 50 items were sampled to serve as field-test items. Figure 1 shows the distribution of the operational item parameters in the pool. The plot for the $b_{i}$ parameters reveals a pool that was slightly on the more difficult side with a mean equal to 0.260 and a standard deviation of 1.267. As the items were originally calibrated using the method of maximum marginal likelihood (MML) estimation, their posterior distributions were taken to be normals with the MML estimates and their standard errors as mean and standard deviation, respectively. From each of the distributions, 500 values were randomly drawn and stored in the system to represent the parameters of the operational items during testing.

**Fig. 5:**
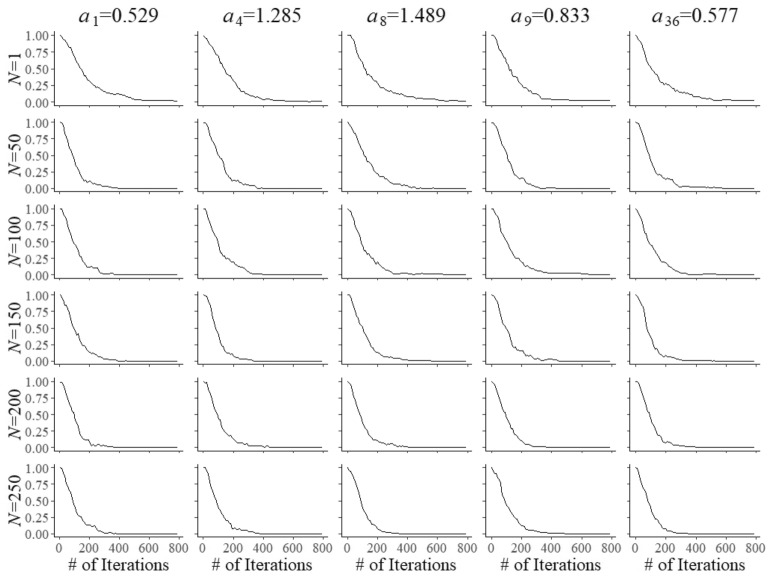
Percentage of replications required to meet the convergence criterion of $\sqrt{\hat{R}}<1.1$ as a function of the number of iterations for field test parameters $a_{{f}}$ of five arbitrary items after $N=1, 50, 10, 50, 150, 200$, and 250 responses

**Fig. 6:**
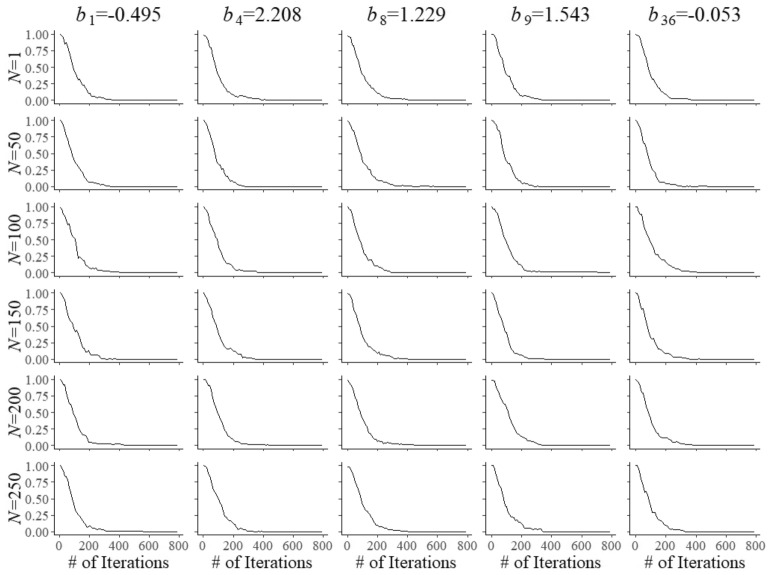
Percentage of replications required to meet the convergence criterion of $\sqrt{\hat{R}}<1.1$ as a function of the number of iterations for field test parameters $b_{{f}}$ of five arbitrary items after $N=1, 50, 10, 50, 150, 200$, and 250 responses

**Fig. 7:**
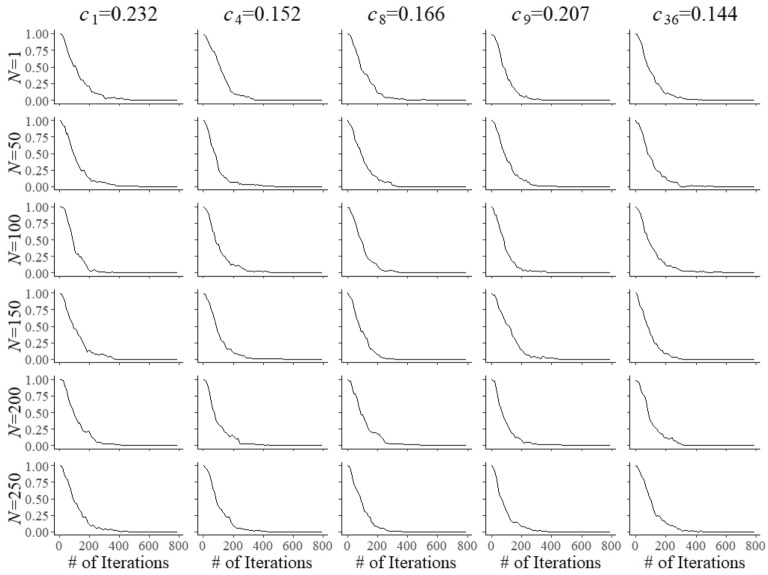
Percentage of replications required to meet the convergence criterion of $\sqrt{\hat{R}}<1.1$ as a function of the number of iterations for field test parameters $c_{{f}}$ of five arbitrary items after $N=1, 50, 10, 50, 150, 200$, and 250 responses

### Simulated Adaptive Testing

The adaptive test consisted of a total of 25 items, 22 of which were operational and three field-test items. As it was unnecessary to hide field-test items from simulated examinees in this study, for practical purposes their position was chosen to be the 21st, 23rd and 25th in the test for each examinee. The test had eight different content specifications modeled as categorical constraints. No quantitative constraints were required, but otherwise the shadow-test model was entirely similar to (23)–(33). The simulated examinees had ability parameter values randomly sampled from the population served by the testing program, which was estimated to be $N(0.26,1.4^{2})$.

The Gibbs sampler for the ability parameter updates consisted of resampling of the 500 posterior draws for the operational item parameters in the system and the MH step in (7)–(11). Burn-in was for 250 iterations, while 500 independent post burn-in draws were saved for the next steps. The sample of 500 was obtained by thinning the post burn-in part of the chain by a factor of ten. These choices have been shown to be conservative for adaptive testing under the 3PL model (van der Linden and Ren 2020). The algorithm started with an initial prior distribution of $N(0.26,1.68^{2})$ for the ability parameter for each of the simulated examinees.

### Item-Calibration Algorithm

The algorithm for item calibration was exactly the version of the Gibbs sampler above with the temporary reparameterization of the field-test parameters and subsequent use of the criterion of $D_{\mathrm{s}}$-optimality in (22). The initial prior distributions of the field-test parameters were normals for $b_{i}$, $\text {ln}(a_{i})$, and $\text {logit}(c_{i})$. As their parameters, the means and SDs for these quantities for the operational item pool were chosen, which were equal to 0.260 (1.520), $-0.396\, (0.582)$, and $-1.414\, (0.528)$, respectively. Because the $D_{\mathrm{s}}$-criterion does not discriminate between items with a common initial prior distribution for their parameters, the simulation started with random assignment of each of the items to five examinees. The calibration process for an item was stopped as soon as it reached the criterion of *N*$=$ 250, 500 or 1,000 examinees. For the 3PL model, these calibration sample sizes are low relative to those typically used for off-line item calibration, especially the two sizes of 250 and 500. Once an item was calibrated, it was treated as an operational item but with a large penalty as weight for its decision variable in the objective function in the shadow-test model. As a consequence, the model tried to avoid selecting the item but at the same time kept the impact of its presence on the relative severeness of the constraints in the model constant during the simulation.

### Simulation Environment

All simulations were run on an i5-6300U 2.4GHz CPU with 16GB of RAM. The simulation framework was written in *R* with the Gibbs samplers coded in *Java*. The shadow tests were assembled making calls to a MIP solver installed on the same machine. Various open-source and commercial solvers are available. Open-source solvers as *lpSolve* and *CBC* (Forrest and Lougee-Heimer 2015) are free to use and can be easily integrated into most simulation frameworks. But commercial solvers as *FICO Xpress* and *IBM CPLEX* allow for significant reduction of solving time without any sacrifice of solution quality. For instance, the *FICO Xpress* solver used in the current simulations has built-in pre-solve algorithms and cut strategies producing solutions to typical problems 10 to 20 times faster than open-source solvers (Meindl and Templ 2012). The following settings were used for the main parameters of this solver: MIPRELSTOP = 1.0E−04, MIPABSSTOP = 0.0, MIPTOL = 5.0E−06, and FEASTOL: 1.0E−06. For a full list of parameters, including their definitions and default values, see the *FICO XPRESS Optimizer Reference Manual*.

### Results

Convergence of the Markov chains for the updates of the field-test parameters is illustrated by the trace plots in Figs. 2, 3 and 4. The plots are for five arbitrary items with different true parameter values after the responses by *N* = 1, 50, 100, 150, 200, and 250 examinees. Of course, after a single examinee the behavior of the chains was rather wild. But with increasing numbers, just as required, they quickly became regular with smaller and smaller posterior uncertainty located at the true values of the parameters.

Convergence of the chains was assessed more analytically using the well-known Gelman-Rubin (1992) diagnostic. In a separate set of simulations, four parallel chains were started from values randomly sampled from the tails of the initial prior distribution for each of the parameters, two from below its mean minus 2SD and the other two from above the mean plus 2SD. The chains were considered to be mixed once a potential scale reduction factor of $\sqrt{\hat{R}}< 1.1$ was reached. The percentages of replications required to meet the criterion as a function of burn-in length for the same five items and numbers of examinees are displayed in Figs. 5, 6 and 7. The results are quite robust. With the exception of the update after $N=1$ examinee, all plots are remarkably similar, even across the three different types of parameters. Just to remain on the safe side, it was decided to run the main simulations with a burn-in length of 800 iterations for the updates of each of the field-test parameters.

**Fig. 8:**
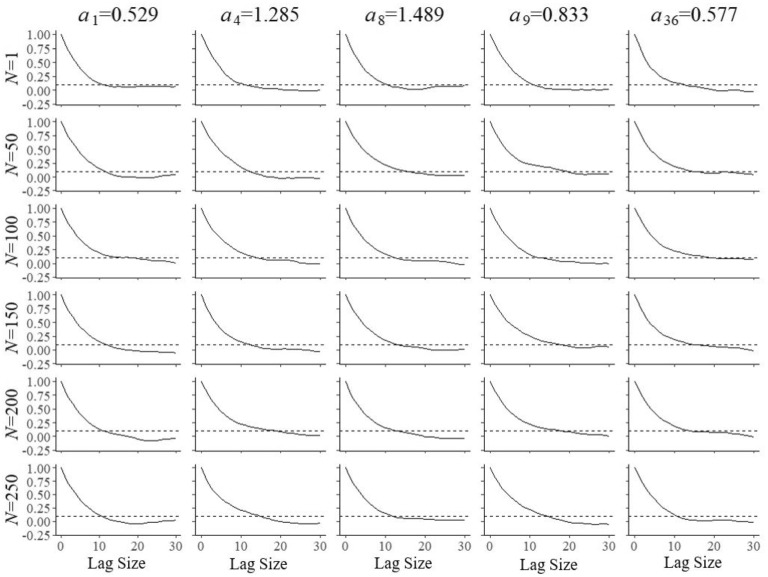
Autocorrelation in the Markov chains as a function of the lag size for field test parameter $a_{{f}}$ of five arbitrary items after $N=1, 50, 10, 50, 150, 200$, and 250 responses

**Fig. 9:**
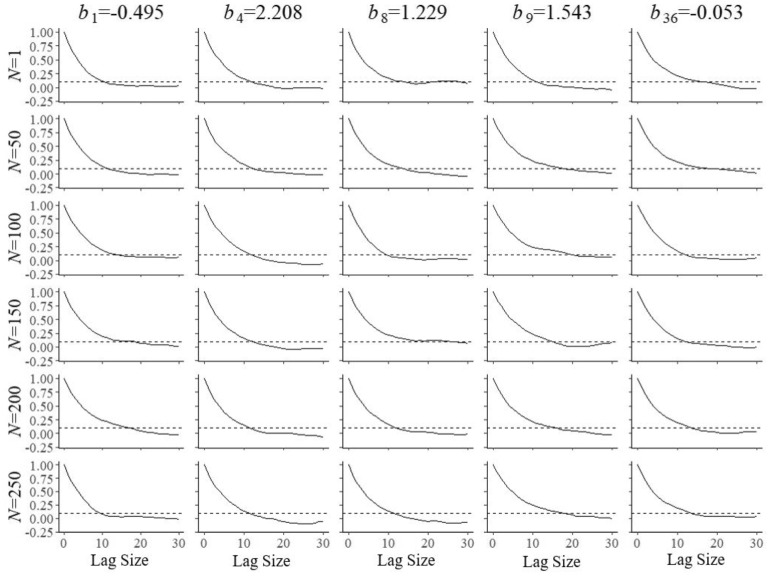
Autocorrelation in the Markov chains as a function of the lag size for field test parameter $b_{{f}}$ of five arbitrary items after $N=1, 50, 10, 50, 150, 200$, and 250 responses

**Fig. 10:**
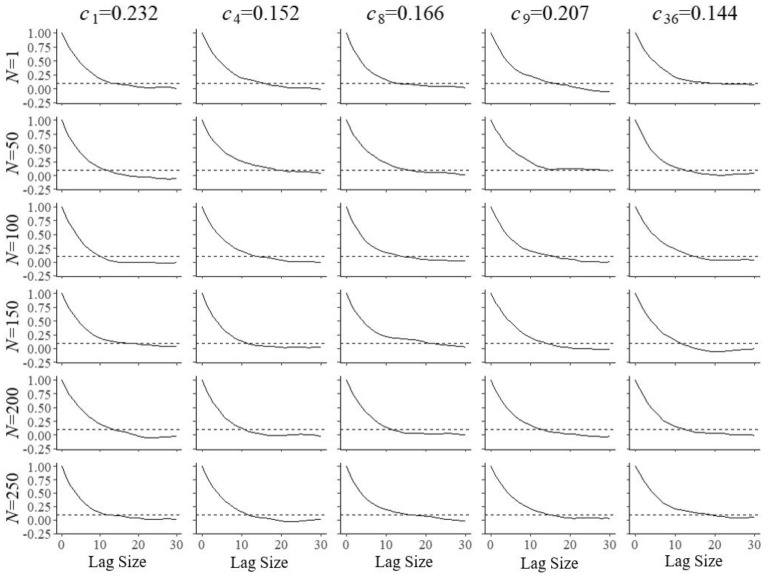
Autocorrelation in the Markov chains as a function of the lag size for field test parameter $c_{{f}}$ of five arbitrary items after $N=1, 50, 10, 50, 150, 200$, and 250 responses

A second set of simulations was run to estimate the autocorrelation of the Markov chains as a function of the lag size during the updates of the field-test parameters. Its results were necessary to obtain samples of independent post burn-in draws. Figures 8, 9 and 10 illustrate the functions estimated from 1,000 replications for the same items and numbers of examinees as in the previous figures. The choice of a lag size of $l = 20$ iterations was large enough to meet the criterion of autocorrelation of $\rho _{l} < 0.1$ for all simulated conditions. The lag size was then used to thin the post burn-in chain to obtain samples of $S=500$ independent draws for the selection of the next item and parameter update. The Monte Carlo error added to standard error of the posterior mean is known to be equal to a factor of $\sqrt{1+1/S}\times 100\%$ (Gelman et al. 2014, Sect. 11.5), which for our choice of $S=500$ amounts to a relative error as small as 0.1%.

**Fig. 11:**
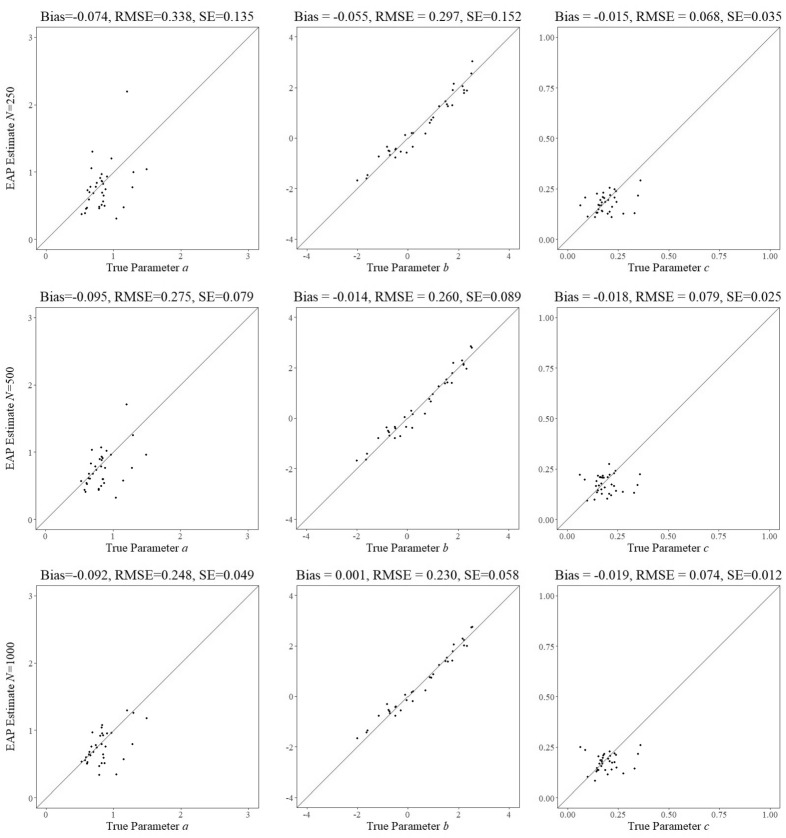
Posterior means versus true values of all field test parameter after $N=250$, 500, and 1,000 responses

**Fig. 12:**
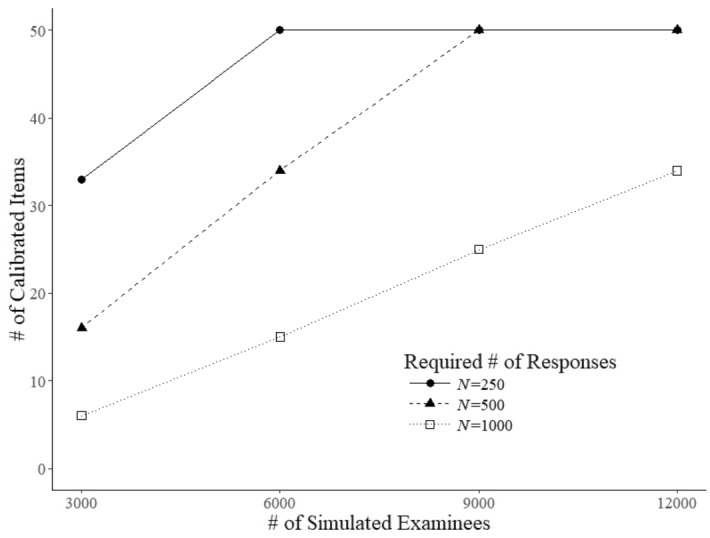
Number of field-test items calibrated as a function of the number of simulated examinees after $N=250$, 500, and 1,000 responses

**Fig. 13:**
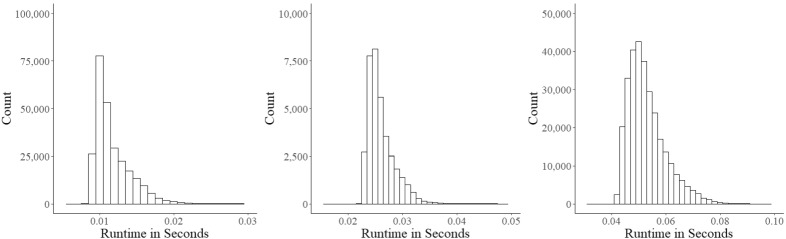
Runtime distributions for the updates of ability parameter $\theta$ (left panel), updates of field-test parameters $\varvec{\xi }_{{f}}$ (middle panel), and calls to the MIP solver with subsequent selection of the next item (right panel) in the main study (12,000 simulated examinees)

The main simulation conducted to evaluate the complete shadow-test approach was for 12, 000 simulated examinees with ability parameters sampled from $N(0.26,1.4^{2})$ and the choice of burn-in length and number of independent post burn-in draws equal to 250 and 500 for the update of the ability parameters and 800 and 500 for the update of the field-test parameters, respectively. Figures 11, 12 and 13 show the EAP estimates of the field-test parameters when the three different calibration sample sizes were reached plotted against their true values. The results for the $b_{{f}}$ and $c_{{f}}$ parameters for a sample size of $N=250$ were already close to those typically seen in fixed-form item calibration for the 3PL model. In fact, the increase in sample size from $N=250$ to $N=1,000$ did hardly have any impact on the accuracy of the estimates for these two parameters. The increase was welcome for the $a_{{f}}$ parameters, however. The larger biases for the smallest sample size for these parameters disappeared with the increase of it, with the exception of a few items with higher true values for the parameter for which the decrease was only minor. The authors checked the simulated runs for these items carefully but have been unable to detect any irregularities or factors that could explain the exceptions. They may just have been the result of the randomness inherent in the procedure.

The criterion of $D_{\mathrm{s}}$-optimality is known to be discriminating with respect to the assignment of items to examinees. Generally, it tends to prefer a few items in the pool, moving to the next few only when their calibration is completed (van der Linden and Ren 2015). The behavior was observed in almost pure form in the current study. Figure 12 displays the number of items that completed the calibration as a function of number of simulated examinees. The functions were nearly perfectly linear for each of the three sample sizes until the maximum number of items was reached. These results point at an item-calibration approach that produces a constant stream of new items ready for operational use by the testing program right from its launch.

### Runtimes

The distributions of the times for the Gibbs sampler to update the ability and field-test parameters and for the calls to the MIP solver with subsequent selection of the next item in the main study with 12,000 examinees are shown in Fig. 13. Their averages (standard deviations) were equal to 0.012 (0.003), 0.026 (0.003), and 0.053 (0.007) s, respectively. These results demonstrate appropriateness of the approach for use in real-world testing.

## Discussion

Key features of the proposed approach to real-time item calibration are embedding of the field-test items in operational adaptive testing in random positions near the end of the test, representation of each of the response model parameters by short vectors of draws from their posterior distributions instead of fixed point estimates, sequential updating of these vector of posterior draws after each new response by two special versions of the Gibbs sampler, use of a well-known optimal design criterion both for the selection of the operational and field-test items, and maintenance of the integrity of the adaptive testing program through a shadow-test approach designed to keep all non-statistical parameters of the program within their bounds. Even for the rather conservative choice of settings for the two Gibbs samplers in our main study, the observed runtimes were fully comparable with those for conventional adaptive testing without any item calibration.

The approach delivers a constant stream of new items ready to replace obsolete items in the pool when their parameter updates have reached the desired degree of statistical precision. The fact that the parameters of these items are estimated directly on the scales in use for the item pool looks especially valuable. It prevents the necessity of separate parameter linking studies after item calibration and promises the best possible scale maintenance through the use of the entire item pool as anchor rather than a few selected items. The expected gains in item parameter stability relative to conventional linking studies have not been evaluated yet and deserve further study.

The current study carefully optimized the settings of the two Gibbs samplers for their application in the proposed shadow-test approach to adaptive testing. Though the settings for their main application in the study reported above were chosen to be conservative and the same choice has been found to work equally well in other applications, including adaptive testing from real-world pools of items calibrated under different polytomous IRT models (Ren et al. 2020), the authors do not claim universal validity. Critical factors not varied in the present study include the composition of the item pool, distribution of the item parameters, the set of test specifications, as well as the length of the test, each of which still has an unknown impact on the behavior of the proposed algorithms. Testing programs interested in an application are recommended to run an additional simulation for their specific environment using our settings as point of departure.

The authors also are aware of fact that the linear results for the speed of item calibration demonstrated in Fig. 12 do not automatically hold for testing programs with continuous item writing and calibration rather than one fully calibrated batch of new items at a time as in the current study. As pointed out by Ren et al. (2017), it is not unlikely for a more continuous environment to find items with less than optimal statistical features being permanently dominated by some of the new items added to the pool. If so, they never complete their calibration, something especially undesirable when they belong to categories in the item pool that have become scarce. The issue brings us to the wider topic of item-pool management and its relation to field-testing of new items. The proposed shadow-test approach offers unique tools that support active management. One option to increase the speed of calibration of items in badly needed categories is using the constraints in (30) to set lower bounds on their presence in the shadow tests. If the reason for lower than expected speed is less favorable statistical features of the field-test items, it is possible to put extra weight on their coefficients in the objective function of the model in (23). Both options can be exercised temporarily when monitoring the developments in the item pool in real time.

As stopping criterion, our study used fixed numbers of responses per item. An alternative criterion is a fixed level of posterior accuracy for the items. As both criteria are monotonically related for each item, we do not expect much different results for the latter. In fact, we expect sensible testing programs to monitor the posterior behavior of the field-test items until a prespecified level has been reached while capping their number of administrations to avoid occasional overexposure. When in this monitoring mode, they may even wish to decide stopping the calibration process for items beginning to show less than satisfactory fit to the response model for their program before either criterion is reached.

## References

[CR1] Berger MPF, van der Linden WJ (2018). Item calibration designs. Handbook of item response theory. Volume 3: Applications.

[CR2] *CBC User Guide*. Retrieved from https://www.coin-or.org/Cbc/.

[CR3] Chang H-H, Ying Z (2009). Nonlinear sequential design for logistic item response models with applications to computerized adaptive tests. The Annals of Statistics.

[CR4] Chen D-S, Batson RG, Dang Y (2010). Applied integer programming.

[CR5] *FICO Xpress Optimizer Reference Manual*. Retrieved from https://www.fico.com/fico-xpress-optimization/docs/latest/solver/optimizer/HTML/GUID-3BEAAE64-B07F-302C-B880-A11C2C4AF4F6.html.

[CR6] FICO. *Xpress optimization*. Retrieved from https://www.fico.com/en/products/fico-xpress-optimization.

[CR7] Forrest, J., & Lougee-Heimer, R. (2015). *CBC user guide*. Retrieved from https://www.coin-or.org/Cbc/.

[CR8] Gelman A, Carlin JB, Stern H, Dunson DB, Vehtari A, Rubin DB (2014). Bayesian data analysis.

[CR9] Gelman A, Rubin DB (1992). Inference from iterative simulation using multiple sequences. Statistical Science.

[CR10] Gilks WR, Richardson S, Spiegelhalter DJ (1996). Markov chain Monte Carlo in practice.

[CR11] Holling H, Schwabe R, van der Linden WJ (2018). Statistical optimal design theory. Handbook of item response theory. Volume 2: Statistical tools.

[CR12] IBM. *CPLEX optimizer*. Retrieved from https://www.ibm.com/analytics/cplex-optimizer.

[CR13] *lp\_solve referenceguide*. Retrieved from http://lpsolve.sourceforge.net/5.5/ [2] C.

[CR14] Meindl, B., & Templ, M. (2012). Analysis of commercial and free and open source solvers for linear optimization problems. *Eurostat and Statistics Netherlands within the project ESSnet on common tools and harmonized methodology for SDC in the ESS*.

[CR15] Ren, H., Choi, S. W., & van der Linden, W. J. (2020). Bayesian adaptive testing with polytomous items. *Behaviormetrika*. 10.1007/s11336-020-09703-8.

[CR16] Ren H, van der Linden WJ, Diao Q (2017). Continuous online item calibration: Parameter recovery and item calibration. Psychometrika.

[CR17] van der Linden WJ (2005). Linear models for optimal test design.

[CR18] van der Linden WJ, van der Linden WJ (2018). Adaptive testing. Handbook of item response theory. Volume 3: Applications.

[CR19] van der Linden, W. J., & Choi, S. W. (2019). Improving item-exposure control in adaptive testing. *Journal of Educational Measurement*. 10.1111/jedm.12254.

[CR20] van der Linden WJ, Reese LM (1998). A model for optimal constrained adaptive testing. Applied Psychological Measurement.

[CR21] van der Linden WJ, Ren H (2015). Optimal Bayesian adaptive design for item calibration. Psychometrika.

[CR22] van der Linden WJ, Ren H (2020). A fast and simple algorithm for Bayesian adaptive testing. Journal of Educational and Behavioral Statistics.

[CR23] Williams HP (2013). Model building in mathematical programming.

